# Electron Microscopy–Based Study of Cannulas for Suspension‐Based Dermal Filler and Biostimulator Application

**DOI:** 10.1111/jocd.70572

**Published:** 2025-11-26

**Authors:** Flavia Radke, Ulrich Gärtner, Anika Seipp, Marina Gorczyza, Stas Wüst

**Affiliations:** ^1^ Vita Aesthetica Berlin Germany; ^2^ Institute of Anatomy and Cell Biology Justus Liebig University Giessen Giessen Germany; ^3^ Dermatologie Mahlow, Blankenfelde‐Mahlow Berlin Germany; ^4^ Z282, Medical Affairs Consulting Beverly Massachusetts USA

**Keywords:** blunt cannula, CaHA, calcium hydroxylapatite, PLLA, poly‐L‐lactic acid, suspension‐based filler

To the Editor,

The use of suspension‐based biositimulators and dermal fillers to activate collagen production is a growing field in esthetic medicine [1]. Poly‐L‐lactic acid (PLLA) and calcium hydroxylapatite (CaHA) are among the most widely used suspension‐based biositimulatory fillers. Both consist of solid microparticles suspended in an aqueous carrier solution or a gel. PLLA is a biodegradable polymer that undergo fibroblast activation and collagen neogenesis for over several months, producing gradual and long‐lasting volume restoration [2]. CaHA combines immediate volumization through its gel matrix with sustained collagen sitimulation as the calcium phosphate microspheres are resorbed [3]. The particle size of these formulations and their heterogeneous rheology make them particularly sensitive to cannula design and lumen geometry, which can influence flow resistance and the risk of clogging during injection.

Initially, these fillers were administered using sharp hypodermic needles; however, there has been a marked shift toward the use of blunt cannulas. Blunt cannulas can reduce the risk of puncturing blood vessels, thereby minimizing hematoma formation [4]. Additionally, when a sufficiently long cannula is used with a fanning technique, the number of entry points can be considerably reduced.

Suspension‐based fillers consist of microparticles (range: 2–150 μm) in an aqueous solution and, when well mixed, generally behave as Newtonian fluids [5, 6]. However, when the particle‐to‐liquid ratio changes—such as when particles stall within the cannula—aggregation can occur, leading to clogging. This can increase the pressure required to inject the filler and may necessitate cannula exchange during the procedure, causing added stress for both the injector and patient.

For this reason, cannulas designed specifically for suspension‐based fillers should meet additional criteria beyond those set by regulatory bodies like the CE, EMA, and FDA. We propose three essential design features: a secure fixation mechanism (e.g., Luer lock) to withstand high pressures, an obstruction‐free inner lumen, and a wide opening. Based on these criteria, we compared 22 gage, 50‐mm cannulas from two leading brands used in Europe: STERiGLIDE by TSK Laboratory International (TSK) and Mirror Soft by Chaeum Pharma (MS).

Both cannula brands feature Luer lock threads, so we focused on an electron microscopic analysis of the exit opening size first (Figure 1). The exit opening size along the tube of the MS cannula measured approximately 1619 μm, while the TSK cannula opening measured 766 μm. Notably, the inner edge of the TSK cannula exhibited jagged edges, unlike the smooth edge of the MS cannula (Figure 1C,D). We then examined the inner wall structure of each cannula (Figure 2). The TSK cannula showed spatter‐like spheres likely molten metal droplets or vaporized alloy condensates, ranging from approximately 0.1 to 8.5 μm, which were absent in the MS cannula.

**FIGURE 1:**
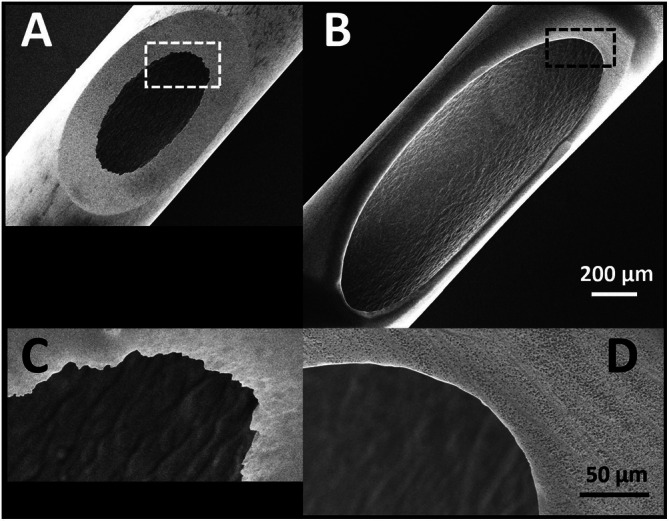
Exit opening comparison of 22 gauge cannulas (TSK cannula: A, C; MS cannula: B, D). Upper panel scale bar: 200 μm, lower panel scale bar: 50 μm.

**FIGURE 2:**
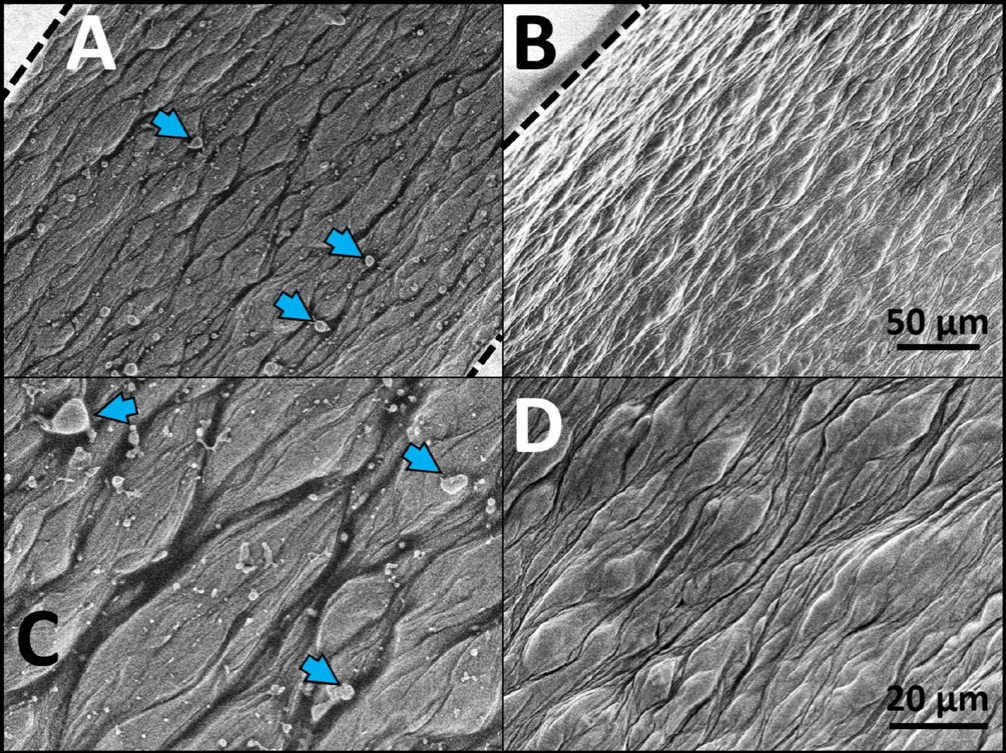
Inner wall comparison of 22 gauge cannulas (TSK cannula: A, C; MS cannula: B, D). Images taken through the exit opening. Arrows indicating spatter‐like spheres. Upper panel scale bar: 50 μm; lower panel scale bar: 20 μm.

While further research is needed, our preliminary findings suggest that the MS cannula may be more suitable for the injection of suspension‐based fillers. Its wider opening could facilitate smoother flow, reducing the likelihood of clogging. In contrast, when using the fanning technique, the jagged edges of the TSK cannula might scrape microscopic parts of connective tissue inside of the cannula causing the cannula to clog. Additionally, the spatter‐like spheres inside the TSK cannula could further contribute to clogging, although their impact depends on their size and quantity further inside of the cannula tube, which was not evaluated in this study. Both cannulas feature Luer lock adapters, ensuring they can withstand high injection pressures.

As a next step, we recommend a larger study to assess flow dynamics and to analyze the condition of the cannulas post‐use. This data, alongside our findings, may help inform the design of cannulas opitimized for suspension‐based filler injections.

## Author Contributions

F.R., M.G., U.G., and S.W. developed the idea and coordinated the project. U.G. and A.S. operated the SEM. S.W. composed the manuscript. All authors contributed significantly to the creation of the final manuscript.

## Ethics Statement

The authors have nothing to report.

## Conflicts of Interest

F.R. is a trainer and consultant for Galderma. M.G. is a consultant for Allergan Aesthetics, Galderma, Merz Aesthetics, and PromaMedical. S.W. is a consultant for Galderma, Lemma Health, and PromaMedical.

## Supporting information


**Data S1:**Supporting Information.

## Data Availability

The data that supports the findings of this study areareis available in the Supporting Information of this article.
