# Supplementary material for: Electron Microscopy–Based Study of Cannulas for Suspension‐Based Dermal Filler and Biostimulator Application
Source: J Cosmet Dermatol. 2025 Nov 26;24(12):e70572. doi: 10.1111/jocd.70572 (PMC12648355; doi:10.1111/jocd.70572)
Supplement: Supplementary file 1 — Data S1:Supporting Information. [file JOCD-24-e70572-s001.docx]

Supplementary Data

Electron Microscopy-Based Study of Cannulas for Suspension-Based Filler Application

# Materials and Methods:

## Microscopy

22 G, 50 mm STERiGLIDE™ cannulas from TSK Laboratory International and 22 G, 50 mm Mirror Soft® from Chaeum Pharma were mounted to aluminum sample trays using double-sided, adhesive carbon tabs and additionally fixed with a conductive carbon adhesive (N650 Planocarbon; all Plano GmbH, Wetzlar, Germany). Afterward, bevels were examined under the scanning electron microscope (XL30; Philips Electron Optics, Eindhoven).

## Image Analysis

The program Image J 1.54g was used to measure the length of the exit opening of the cannulas. Due to strict regulations enforced by the European Medicines Agency in the field of medical devices, the authors assumed that the variation rates between cannulas from one manufacturer are minimal to none. Based on this assumption the authors analyzed only an *n* of 1 of each cannula.
